# Performance Analysis and Optimization for Irreversible Combined Carnot Heat Engine Working with Ideal Quantum Gases

**DOI:** 10.3390/e23050536

**Published:** 2021-04-27

**Authors:** Lingen Chen, Zewei Meng, Yanlin Ge, Feng Wu

**Affiliations:** 1 Institute of Thermal Science and Power Engineering, Wuhan Institute of Technology, Wuhan 430205, China; geyali9@hotmail.com (Y.G.); 13006338568@163.com (F.W.); 2 School of Mechanical & Electrical Engineering, Wuhan Institute of Technology, Wuhan 430205, China; 3 College of Power Engineering, Naval University of Engineering, Wuhan 430033, China; mengzw94@163.com

**Keywords:** finite-time thermodynamics, Carnot heat engine, irreversible combined cycle, ideal quantum gas, power output, thermal efficiency

## Abstract

An irreversible combined Carnot cycle model using ideal quantum gases as a working medium was studied by using finite-time thermodynamics. The combined cycle consisted of two Carnot sub-cycles in a cascade mode. Considering thermal resistance, internal irreversibility, and heat leakage losses, the power output and thermal efficiency of the irreversible combined Carnot cycle were derived by utilizing the quantum gas state equation. The temperature effect of the working medium on power output and thermal efficiency is analyzed by numerical method, the optimal relationship between power output and thermal efficiency is solved by the Euler-Lagrange equation, and the effects of different working mediums on the optimal power and thermal efficiency performance are also focused. The results show that there is a set of working medium temperatures that makes the power output of the combined cycle be maximum. When there is no heat leakage loss in the combined cycle, all the characteristic curves of optimal power versus thermal efficiency are parabolic-like ones, and the internal irreversibility makes both power output and efficiency decrease. When there is heat leakage loss in the combined cycle, all the characteristic curves of optimal power versus thermal efficiency are loop-shaped ones, and the heat leakage loss only affects the thermal efficiency of the combined Carnot cycle. Comparing the power output of combined heat engines with four types of working mediums, the two-stage combined Carnot cycle using ideal Fermi-Bose gas as working medium obtains the highest power output.

## 1. Introduction

Combining with thermodynamics, heat transfer, and fluid mechanics, finite-time thermodynamics (FTT) has been widely applied in the performance analyses and optimizations of various heat engines (HEs), refrigerators and heat pump cycles, and many meaningful results have been obtained. At present, FTT is an important part of modern thermodynamics [1,2,3,4,5,6,7,8,9,10,11,12,13,14,15,16,17,18,19,20,21,22,23,24,25,26,27,28,29,30,31,32,33,34,35,36,37].

Using FTT, many scholars have studied from single cycles to multi-stage combined cycles with various types of traditional working mediums (WMs). Rubin and Andresen [38] first studied the two-stage endoreversible combined HE with intermediate heat reservoirs in 1982 and pointed out that when the combined HE operated between the fixed hot reservoir and the fixed cold reservoir, the efficiency at the maximum power (EMP) of the single-stage cycle was the same as that of the multi-stage cycle, and the two efficiencies were equal to Curzon-Ahlborn efficiency. After that, Chen and Yan [39] derived the optimal efficiency and heating supply rate of an endoreversible combined Carnot cycle without intermediate heat reservoirs. Wu [40,41] analyzed the influence of the types of WMs on the endoreversible combined HE. The results showed that compared with a single WM, the combined cycle using different WMs could effectively expand the temperature difference between the hot reservoir and cold reservoir. Hence, it could improve the output performance of the combined HE. In order to obtain a more realistic combined cycle model, Chen [42] established a generalized irreversible combined HE model considering thermal resistance, heat leakage loss, and internal irreversibility. Then, different performance evaluations and research methods have been taken into account, including power output [43,44,45,46,47] and entransy loss [48,49]. Iyyappan and Johal [50] analyzed the linear irreversible two-stage combined HE with low dissipation. Under the condition of tight coupling, each stage HE presented low dissipation behavior, that is, entropy generation was inversely proportional to the duration of the process.

With the development of new technology and the demand for energy, micro-scale energy conversion devices have gradually attracted the interest of scholars. A great deal of literature has applied FTT theory to study the thermodynamic performance of HEs such as Brownian motor [51,52] and micro-/nanoscaled energy conversion systems [53,54]. FTT theory is also extended to the study of quantum heat engines (QHE). Since 1984, combined with quantum mechanics and FTT, Kosloff [55,56] established a QHE model with a finite heat transfer rate and studied the power and efficiency of the QHE using a harmonic oscillator system [55,56] and spin-1/2 system [57] as WMs, respectively. Sisman and Saygin [58,59,60,61] employed ideal quantum gases as the WMs and applied the WMs to establish the Ericsson cycle [58], Carnot cycle [59], Stirling cycle [60], and Otto cycle [61] models. Considering the effect of quantum degeneracy, Lin and Chen [62] established the Brayton Fermi cycle and focused on the output work and thermal efficiency of the system. Considering the influence of thermal resistance and internal irreversibility, Wang et al. [63,64,65] analyzed the Otto cycle and Brayton cycle with Bose gas and obtained the relationships among the power, efficiency, and the optimal pressure ratio. Açikkalp and Caner [66,67] analyzed the performances of the Dual cycle and Brayton cycle with quantum gas, and deduced performance indexes such as work, exergy output, ecological function, thermal efficiency, and exergy efficiency. In the past years, FTT theory has been widely used to study all kinds of QHEs, including Carnot [68,69,70], Otto [71,72,73], Stirling [74], and Brayton [75] QHEs, from the reversible cycle, endoreversible cycle to the irreversible cycle. Different optimization objective functions, from power, efficiency to ecological function, and thermo-economic performance in single-stage quantum thermodynamic cycles, have been studied widely [76,77,78,79,80,81,82,83,84,85,86,87,88,89].

The research results on classical HEs have shown that a combined cycle can further improve the energy utilization rate and avoid the waste of energy [38,39,40,41]. The researches on the combined cycle have mostly focused on the classical working medium. However, with the development of lasers, nanodevices, and cryogenic refrigeration devices, these will involve energy conversion and energy loss. Generally, the energy conversion processes are analyzed by simplified theoretical models such as quantum HE and quantum refrigerator. Through these theoretical models, scholars can analyze and optimize their power, efficiency and the optimal working range, and so on. Then these results further guide the practical applications. There are many works, including theoretical studies and experimental studies [89,90,91,92], focused on single-stage single-atom HEs and QHEs, but little research on combined QHEs.

If building a combined cycle with quantum WM, would the result be the same? Would there be new results? For the quantum combined HE, Meng et al. [93] studied the combined quantum harmonic HE with an intermediate heat reservoir by using FTT theory. The results showed that the combined HE had three operating modes with different temperatures of WM, and the improving extents of power and efficiency linearly increased with the number of stages. For thermal Brownian engines, Qi et al. [94,95] studied the combined thermal Brownian HE [94] and refrigerator [95] by using FTT theory. To date, the combined HE using ideal quantum gas as WM has not been studied in the open literature. Based on the Refs. [58,93], an irreversible combined cycle model with ideal quantum gas will be established in this paper, and the output power and efficiency of the combined HE will be analyzed and optimized. Quantum gas will be used as WM for the first time to establish an irreversible combined cycle by using FTT theory. It is important and valuable to extend the application of FTT theory and to study the characteristics of an irreversible combined cycle.

## 2. Theoretical Model for Heat Engine with Quantum Gas

The basic thermodynamic parameters of quantum gas will be introduced. Then, the combined HE model will be established in this section.

### 2.1. The Physical Characteristics for Quantum Gas

According to the theory of ideal quantum gas, the gas state equation is described as [58,96]

(1) $$p=n_{q}kT\cdot CF_{q}\left(z\right)$$

where p denotes gas pressure, $n_{q}$ denotes number density, k denotes Boltzmann’s constant, T denotes gas temperature, and $CF_{q}\left(z\right)$ denotes correction factor.

For Bose gas and Fermi gas, the definitions of the number density of gas particles and correction factor are different due to the statistical description. The number density of gas particles is expressed as, respectively,

(2) $$n_{q}=n_{B}=\frac{N-N_{0}}{V}=g\lambda^{-3}g_{3/2}\left(z\right)$$

(3) $$n_{q}=n_{F}=\frac{N}{V}=g\lambda^{-3}f_{3/2}\left(z\right)$$

where N denotes the total number of particles, $N_{0}$ denotes the number of particles of Bose gas in a condensed state, V denotes the volume, g denotes the number of possible spin orientations, $\lambda =h/\sqrt{2\pi mkT}$ denotes the mean thermal wavelength, h denotes Planck’s constant, m denotes the rest mass of a gas particle, $z=e^{\mu /kT}$ denotes the fugacity, μ denotes the chemical potential, $f_{l}\left(z\right)=\frac{1}{\Gamma \left(l\right)}\int_{0}^{\infty }\frac{x^{l-1}}{z^{-1}e^{x}+1}dx$ denotes the Fermi integral, $g_{l}\left(z\right)=\frac{1}{\Gamma \left(l\right)}\int_{0}^{\infty }\frac{x^{l-1}}{z^{-1}e^{x}-1}dx$ denotes the Bose integral, and Γ(l) denotes Gamma function.

The correction factors of Bose gas and Fermi gas are given by, respectively

(4) $$CF_{q}\left(z\right)=CF_{B}\left(z\right)=g_{5/2}\left(z\right)/g_{3/2}\left(z\right)$$

(5) $$CF_{q}\left(z\right)=CF_{F}\left(z\right)=f_{5/2}\left(z\right)/f_{3/2}\left(z\right)$$

The corresponding expressions of internal energy and entropy for quantum gas are denoted as, respectively,

(6) $$U=\frac{3}{2}NkT\cdot CF_{q}\left(z\right)$$

(7) $$S=Nk\left[\frac{5}{2}CF_{q}\left(z\right)-\ln\left(z\right)\right]$$

The above equations are the basic thermodynamic parameters of the quantum gas. In the following section, utilizing the thermodynamic characteristics of the above quantum gas, the output performance of the quantum combined HE will be analyzed and the optimal relationship between power and thermal efficiency will be solved.

### 2.2. The Model of Combined Carnot Cycle with Quantum Gas

**Figure 1:**
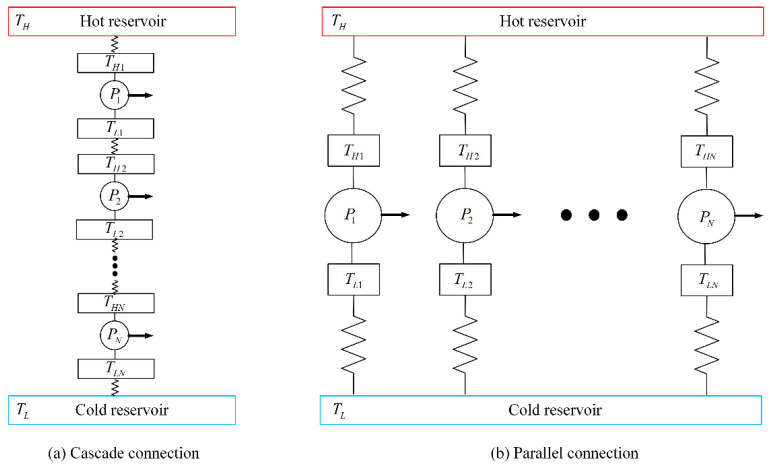
Schematic diagram of two types of combined HE.

The combined HE can be defined as one that consists of several single-stage HEs, which have some correlations and work together. According to the combined forms, it can be classified as parallel connection, cascade connection, etc., and according to the number of stages, it can be classified as a two-stage cycle or multi-stage cycle. Figure 1 shows a schematic of two types of combined HEs.

In this paper, a two-stage cascade Carnot cycle with quantum gas was studied. The operation way of two Carnot sub-cycles is that the top sub-cycle absorbs heat from the hot reservoir, outputs power, and then exhausts heat to the bottom sub-cycle, and the bottom sub-cycle absorbs heat from the top sub-cycle, outputs power, and then exhausts heat to cold reservoir. Through multi-stage utilization of the energy from the hot reservoir, the total available temperature range between hot reservoir and cold reservoir can be expanded, thus, the combined cycle can improve the power and thermal efficiency.

**Figure 2:**
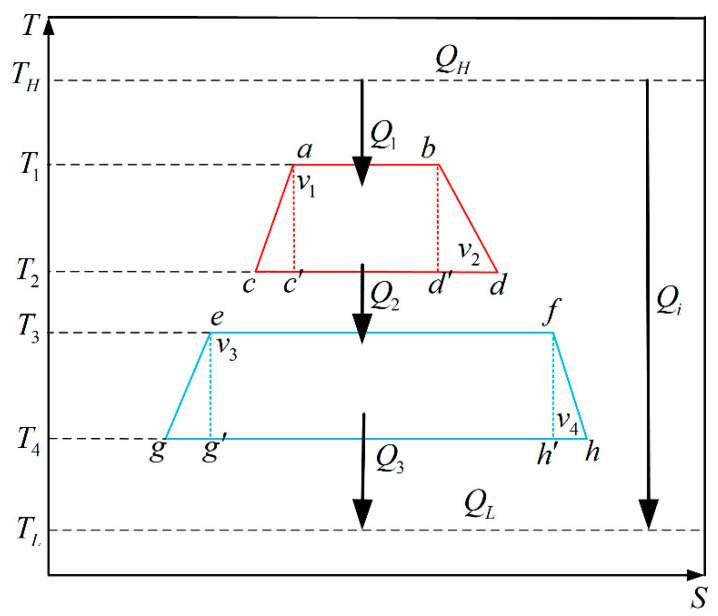
The temperature–entropy diagram of two-stage combined Carnot HE with quantum gas.

To obtain the specific performance of the combined HE with quantum gas, an irreversible combined Carnot cycle was utilized as an example in this paper. Figure 2 shows the temperature-entropy diagram of the combined Carnot cycle. For the combined cycle, the WMs of two sub-cycles were separated by a heat conduction material. The WM of the bottom sub-cycle is used as the cold reservoir ($T_{3}$) of the top sub-cycle, and the WM of the top sub-cycle was used as the hot reservoir ($T_{2}$) of the bottom sub-cycle. Therefore, the top sub-cycle operates between hot reservoir ($T_{H}$) and cold reservoir ($T_{3}$). The temperatures of WM in the isothermal expansion process and isothermal compression process are $T_{1}$ and $T_{2}$, respectively.

The bottom sub-cycle operates between the hot reservoir ($T_{2}$) and cold reservoir ($T_{L}$). The temperatures of WM in the isothermal expansion process and isothermal compression process are $T_{3}$ and $T_{4}$, respectively. In the Figure 2, v=V/N is the average volume that a quantum gas particle occupies.

In the quantum regime, in order to simplify the model, the heat transfer of gas is supposed to obey Newton’s Law [63,97], the amount of absorbing heat from the hot reservoir can be denoted as

(8) $$Q_{1}=\alpha_{1}A_{1}\left(T_{H}-T_{1}\right)t_{1}$$

where $\alpha_{1}$ denotes heat transfer coefficient, $A_{1}$ denotes heat transfer area, and $t_{1}$ denotes process time.

There is no intermediate heat reservoirs for the two sub-engines. Therefore, the amount of heat transfer from the low-temperature WM of the top sub-cycle to the high-temperature WM of the bottom sub-cycle can be expressed as

(9) $$Q_{2}=\alpha_{2}A_{2}\left(T_{2}-T_{3}\right)t_{2}$$

Correspondingly, the amount of heat transfer between the bottom sub-cycle and cold reservoir is

(10) $$Q_{3}=\alpha_{3}A_{3}\left(T_{4}-T_{L}\right)t_{3}$$

There is heat leakage loss between the hot reservoir and cold reservoir, which can be expressed as

(11) $$Q_{i}=C_{i}\left(T_{H}-T_{L}\right)\tau$$

where τ is the cycle period of the combined heat engine and $C_{i}$ is the heat leakage loss coefficient.

The two sub-cycles are Carnot cycles, and the times of adiabatic compression processes and adiabatic expansion processes are negligible. In addition, to keep the two sub-cycles operate synchronized, the two cycle periods should be equal, that is $\tau =t_{1}+t_{2}=t_{2}+t_{3}$. Hence, the time consumed in the isothermal absorbing heat process of the top sub-cycle should be equal to the time consumed in the isothermal exhausting heat process of the bottom sub-cycle, that is

(12) $$t_{1}=t_{3}$$

The internal irreversibilities of the two sub-cycles are given by, respectively

(13) $$D_{1}=\frac{Q_{2}}{Q_{2}^{\prime }}=\frac{T_{2}\left(S_{d}-S_{c}\right)}{T_{2}\left(S_{d^{\prime }}-S_{c^{\prime }}\right)}=\frac{S_{d}-S_{c}}{S_{b}-S_{a}}$$

(14) $$D_{2}=\frac{Q_{3}}{Q_{3}^{\prime }}=\frac{T_{3}\left(S_{h}-S_{g}\right)}{T_{3}\left(S_{h^{\prime }}-S_{g^{\prime }}\right)}=\frac{S_{h}-S_{g}}{S_{f}-S_{e}}$$

where $Q_{2}^{\prime }$ and $Q_{3}^{\prime }$ are the amounts of exhausting heat under the endoreversible conditions in top sub-cycle and bottom sub-cycle, respectively; $Q_{2}$ and $Q_{3}$ are the amounts of exhausting heat under the irreversible conditions in top sub-cycle and bottom sub-cycle, respectively; $S_{a}$, $S_{b}$, $S_{c}$ and $S_{d}$ denote entropies of four state points (a, b, c and d) under the irreversible conditions in top sub-cycle; $S_{c^{\prime }}$ and $S_{d^{\prime }}$ denote entropies of two state points ($c^{\prime }$ and $d^{\prime }$) under the reversible conditions in top sub-cycle; $S_{e}$, $S_{f}$, $S_{g}$ and $S_{h}$ denote entropies of four state points (e, f, g and h) under the irreversible conditions in bottom sub-cycle; as well as $S_{g^{\prime }}$ and $S_{h^{\prime }}$ denote entropies of two state points ($g^{\prime }$ and $h^{\prime }$) under the reversible conditions in bottom sub-cycle (see Figure 2).

To further simplify the internal irreversibilities of the two sub-cycles, the entropy ratios in adiabatic processes of two sub-cycles are defined as, respectively,

(15) $$\phi_{1}=S_{a}/S_{c}$$

(16) $$\phi_{2}=S_{d}/S_{b}$$

(17) $$\phi_{3}=S_{e}/S_{g}$$

(18) $$\phi_{4}=S_{h}/S_{f}$$

Combining Equation (7) with Equations (13)–(18) yields the internal irreversibilities of the two sub-cycles are, respectively;

(19) $$D_{1}=\frac{S_{d}-S_{a}/\phi_{1}}{S_{d}/\phi_{2}-S_{a}}=\frac{\frac{5}{2}CF_{q}\left(z_{d}\right)-\ln\left(z_{d}\right)-\left[\frac{5}{2}CF_{q}\left(z_{a}\right)-\ln\left(z_{a}\right)\right]/\phi_{1}}{\left[\frac{5}{2}CF_{q}\left(z_{d}\right)-\ln\left(z_{d}\right)\right]/\phi_{2}-\left[\frac{5}{2}CF_{q}\left(z_{a}\right)-\ln\left(z_{a}\right)\right]}$$

(20) $$D_{2}=\frac{S_{h}-S_{e}/\phi_{3}}{S_{h}/\phi_{4}-S_{e}}=\frac{\frac{5}{2}CF_{q}\left(z_{h}\right)-\ln\left(z_{h}\right)-\left[\frac{5}{2}CF_{q}\left(z_{e}\right)-\ln\left(z_{e}\right)\right]/\phi_{3}}{\left[\frac{5}{2}CF_{q}\left(z_{h}\right)-\ln\left(z_{h}\right)\right]/\phi_{4}-\left[\frac{5}{2}CF_{q}\left(z_{e}\right)-\ln\left(z_{e}\right)\right]}$$

It can be seen from Equations (19) and (20) that only when all the entropy ratios in adiabatic processes of two sub-cycles meet $\phi_{1}=\phi_{2}=\phi_{3}=\phi_{4}=1$, the internal irreversibilities of the two sub-cycles are $D_{1}=1$ and $D_{2}=1$, and the irreversible combined HE cycle is the endoreversible one.

In the combined HE, both absorbing heat and exhausting heat are isothermal processes. The amounts of exchanging heat are given by

(21) $$Q_{1}=Q_{ab}=T_{1}\left(S_{b}-S_{a}\right)=T_{1}\left(S_{d}/\phi_{2}-S_{a}\right)$$

(22) $$Q_{2}=Q_{cd}=T_{2}\left(S_{d}-S_{c}\right)=T_{2}\left(S_{d}-S_{a}/\phi_{1}\right)$$

(23) $$Q_{2}=Q_{ef}=T_{3}\left(S_{f}-S_{e}\right)=T_{3}\left(S_{h}/\phi_{4}-S_{e}\right)$$

(24) $$Q_{3}=Q_{gh}=T_{4}\left(S_{h}-S_{g}\right)=T_{4}\left(S_{h}-S_{e}/\phi_{3}\right)$$

Combining Equation (22) with Equation (23) yields

(25) $$\;T_{2}\left(S_{d}-S_{a}/\phi_{1}\right)-T_{3}\left(S_{h}/\phi_{4}-S_{e}\right)=0$$

Combining Equations (8), (10) and (12) with Equations (21) and (24) yields:

(26) $$\frac{T_{1}\left(S_{d}/\phi_{2}-S_{a}\right)}{\alpha_{1}A_{1}\left(T_{H}-T_{1}\right)}=\frac{T_{4}\left(S_{h}-S_{e}/\phi_{3}\right)}{\alpha_{3}A_{3}\left(T_{4}-T_{L}\right)}$$

(27) $$\alpha_{3}A_{3}T_{1}\left(T_{4}-T_{L}\right)\cdot \left(S_{d}/\phi_{2}-S_{a}\right)-\alpha_{1}A_{1}T_{4}\left(T_{H}-T_{1}\right)\cdot \left(S_{h}-S_{e}/\phi_{3}\right)=0$$

Equations (25) and (27) are the necessary conditions for sustaining operation of the combined HE.

## 3. The Output Performance of Combined Heat Engine

Combining Equations (8)–(10) and (12) with Equations (21) and (24), the cycle period of combined HE is given by

(28) $$\tau =t_{1}+t_{2}=t_{2}+t_{3}=\frac{T_{1}\left(S_{d}/\phi_{2}-S_{a}\right)}{\alpha_{1}A_{1}\left(T_{H}-T_{1}\right)}+\frac{T_{2}\left(S_{d}-S_{a}/\phi_{1}\right)}{\alpha_{2}A_{2}\left(T_{2}-T_{3}\right)}$$

According to Equations (8)–(24), the power and thermal efficiency of the combined HE are denoted as, respectively,

(29) $$P=P_{1}+P_{2}=\frac{Q_{1}-Q_{2}}{\tau }+\frac{Q_{2}-Q_{3}}{\tau }=\frac{Q_{1}-Q_{3}}{\tau }=\left[T_{1}\left(S_{d}/\phi_{2}-S_{a}\right)-T_{4}\left(S_{h}-S_{e}/\phi_{3}\right)\right]\tau^{-1}$$

(30) $$\eta =\frac{Q_{H}-Q_{L}}{Q_{H}}=\frac{Q_{1}-Q_{3}}{Q_{1}+Q_{i}}=\frac{T_{1}\left(S_{d}/\phi_{2}-S_{a}\right)-T_{4}\left(S_{h}-S_{e}/\phi_{3}\right)}{T_{1}\left(S_{d}/\phi_{2}-S_{a}\right)+C_{i}\left(T_{H}-T_{L}\right)\tau }$$

Substituting Equations (19) and (20) into Equation (25) yields

(31) $$\frac{S_{h}-S_{e}/\phi_{3}}{S_{d}/\phi_{2}-S_{a}}=D_{1}D_{2}\frac{T_{2}}{T_{3}}$$

Using Equations (19), (20), and (31), the power and thermal efficiency can be rewritten as, respectively,

(32) $$P=\frac{T_{1}T_{3}-D_{1}D_{2}T_{2}T_{4}}{\frac{T_{1}T_{3}}{\alpha_{1}A_{1}\left(T_{H}-T_{1}\right)}+\frac{T_{2}T_{3}D_{1}}{\alpha_{2}A_{2}\left(T_{2}-T_{3}\right)}}$$

(33) $$\eta =\left(1-D_{1}D_{2}\frac{T_{2}}{T_{1}}\cdot \frac{T_{4}}{T_{3}}\right)\cdot \frac{1}{1+Ζ}$$

where $Ζ=C_{i}\left(T_{H}-T_{L}\right)\cdot \left\{1/\left[\alpha_{1}A_{1}\left(T_{H}-T_{1}\right)\right]+T_{2}D_{1}/\left[\alpha_{2}A_{2}T_{1}\left(T_{2}-T_{3}\right)\right]\right\}$.

According to Equations (32) and (33), the power and thermal efficiency of the combined HE is determined by the temperature of WM in two sub-cycles, and there are four variables ($T_{1}$,$T_{2}$,$T_{3}$ and $T_{4}$). In fact, the four variables are not independent variables. Combined with Equations (25) and (27), it can be known that when $T_{1}$ and $T_{2}$ is given, $T_{3}$ and $T_{4}$ can be solved by the two equations.

In general, the thermal conductance distribution is also an optimization variable. Setting $k_{a}=\alpha_{3}A_{3}/(\alpha_{1}A_{1})$, Equations (25) and (27) can be rewritten as, respectively,

(34) $$f_{1}\left(T_{3},T_{4}\right)=T_{2}\left(S_{d}-S_{a}/\phi_{1}\right)-T_{3}\left(S_{h}/\phi_{4}-S_{e}\right)=0$$

(35) $$f_{2}\left(T_{3},T_{4},k_{a}\right)=k_{a}T_{1}\left(T_{4}-T_{L}\right)\cdot \left(S_{d}/\phi_{2}-S_{a}\right)-T_{4}\left(T_{H}-T_{1}\right)\cdot \left(S_{h}-S_{e}/\phi_{3}\right)=0$$

**Figure 3:**
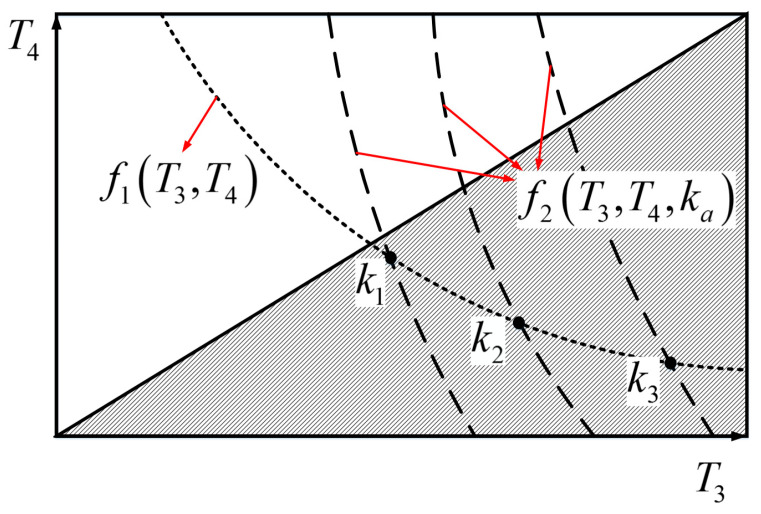
The constraint functions (the shadow area in the figure is the feasible temperature range for normal operation).

A schematic of two constraint functions is depicted in Figure 3, where the curve (dot line) $f_{1}\left(T_{3},T_{4}\right)$ is the feasible solution of Equation (34) and the curve (dashed line) $f_{2}\left(T_{3},T_{4},k_{a}\right)\;$ is the feasible solution of Equation (35). When $k_{a}$ change, $f_{1}\left(T_{3},T_{4}\right)$ and $f_{2}\left(T_{3},T_{4},k_{a}\right)\;$ will intersect at different points. That is, for a set of values ($T_{1}$,$T_{2}$ and $k_{a}$), $T_{3}$ and $T_{4}$ can be obtained according to the intersection point of $f_{1}\left(T_{3},T_{4}\right)$ and $f_{2}\left(T_{3},T_{4},k_{a}\right)\;$. Then, the operating temperatures of the combined HE are obtained. At the same time, for the HE, the temperatures of WM are required to meet $T_{3}>T_{4}$, that is, the shadow area in the figure is feasible temperature range for normal operation.

When $T_{1}$ and $T_{2}$ is given, the solutions of $T_{3}$ and $T_{4}$ are determined by $k_{a}$. When $k_{a}$ is taken for different values, the corresponding temperatures ($T_{3}$ and $T_{4}$) of WM vary and the output performance of the combined HE is also different. Therefore, the local optimal solution of the performance parameters can be obtained through optimizing $k_{a}$. For example, when the output power is taken as the performance evaluation, the different temperatures ($T_{3}$ and $T_{4}$) of WM in the bottom sub-cycle can be obtained through optimizing $k_{a}$ for given temperatures ($T_{1}$ and $T_{2}$) of WM in the top sub-cycle. Therefore, the thermal conductance distribution is chosen as $k_{a}=k_{m}$ that makes power maximize. Under this condition, the corresponding temperatures ($T_{1}$,$T_{2}$,$T_{3}$ and $T_{4}$) of WM are taken as the local optimal solution of the combined heat engine.

The above method only obtains the local optimal solution of the combined HE for each set of temperatures of WM ($T_{1}$, $T_{2}$, $T_{3}$ and $T_{4}$). In fact, there are many parameters to evaluate the performance of HEs, such as maximum power and maximum efficiency, but both cannot be reached at the same time (refer to Figure 6). In practical work, the performance of a HE deviates inevitably from the maximum power and maximum efficiency. In order to balance the contradiction between maximum power and maximum efficiency, the appropriate performance parameters need to be selected. In this paper, the power with constraint of efficiency is selected as the performance index, aiming to obtain the local maximum power (optimal performance) of the heat engine when the efficiency is given or constrained. In order to obtain the global optimal power at a given thermal efficiency, the Lagrangian function method is introduced. Utilizing the constraint Equations (34) and (35), the Lagrangian function can be established as

(36) $$L=P+\lambda_{1}\eta +\mu_{1}f_{1}\left(T_{3},T_{4}\right)+\mu_{2}f_{2}\left(T_{3},T_{4},k_{m}\right)\;$$

In Equation (36), the Lagrange multiplier ($\lambda_{1}$) is introduced to represent the maximum power value under the given efficiency value ($P_{\max,\eta =\eta_{s}}$). In the relationship between optimal power and efficiency, the maximum power and its corresponding efficiency are global optimum point and there is only one point. But in practice, when the efficiency changes, there will be a corresponding local optimal power output. That is, the power output cannot always reach the maximum value. So the Lagrange multiplier is introduced to obtain the relationship between the optimal power and efficiency. $\mu_{1}$ and $\mu_{2}$ are the constraints to ensure the normal operation of the combined heat engine.

Combined with Equations (34) and (35) and Euler-Lagrange equations

(37) $$\partial L/\partial T_{1}=0,\partial L/\partial T_{2}=0$$

the optimum relationship of $T_{1}$ and $T_{2}$ is obtained, and then the optimal relationship of power versus thermal efficiency is also obtained. Since the explicit expressions of temperatures of WM cannot be solved from Equations (34) and (35), the Euler-Lagrange equations are difficult to derive the analytical solution. Therefore, a numerical method is employed to solve the optimal solution of the objective function in the following section.

Firstly, the efficiency is set as a value, of which range is $0<\eta_{s}<\eta_{\max}$. The analyzing process of maximum efficiency ($\eta_{\max}$) will be given in Equation (39) in Section 4.2. Then, numerical methods (enumeration method or Newton iteration method) are used to solve Equation (37), so as to obtain the working conditions of the combined cycle at the maximum power output under the given efficiency ($P_{\max,\eta =\eta_{s}}$). That is, the corresponding temperatures ($T_{1}$,$T_{2}$,$T_{3}$ and $T_{4}$) of WM are obtained by solving Equation (37). Then, it is substituted into Equation (32) to obtain the optimal power value at a given efficiency ($P_{\max,\eta =\eta_{s}}$). By repeating the above steps, the relationship between the optimal output power ($P_{\max,\eta =\eta_{s}}$) and the corresponding efficiency can be obtained, and the corresponding working conditions can also be obtained.

## 4. The General Performance of Power Output and Thermal Efficiency

The general and optimal performances of the combined cycle will be discussed in this section.

### 4.1. The General Combined Cycle for Multi-Stage Endoreversible Carnot QHE

To study the specific output performance of the combined HE, Fermi gas (${}^{3}\mathrm{He}$) and Bose gas (${}^{4}\mathrm{He}$) are selected as WMs [97]. The temperatures of heat reservoirs are set as $T_{H}=90K$ and $T_{L}=10K$, respectively. The minimum average volume and maximum average volume of top sub-cycle are set as $v_{1}=1.1\times 10^{-29}\;m^{3}$ and $v_{2}=1\times 10^{-28}\;m^{3}$, respectively. The minimum average volume and the maximum average volume of the bottom sub-cycle are set as $v_{3}=1\times 10^{-29}\;m^{3}$ and $v_{4}=1.1\times 10^{-28}\;m^{3}$, respectively. It is assumed that all the entropy ratios are all equal, that is $\phi_{1}=\phi_{2}=\phi_{3}=\phi_{4}=\phi$. When the entropy ratio meet ϕ=1, the internal irreversibilities of the two sub-cycles are $D_{1}=D_{2}=1$ and the combined cycle is the endoreversible one. Therefore, the combined cycle can be directly considered as an endoreversible cycle in the following analysis when the entropy ratio meets ϕ=1. Based on the established cycle model and the given parameters, the output power and corresponding thermal efficiency of the combined HE will be analyzed and optimized in the following section.

**Figure 4:**
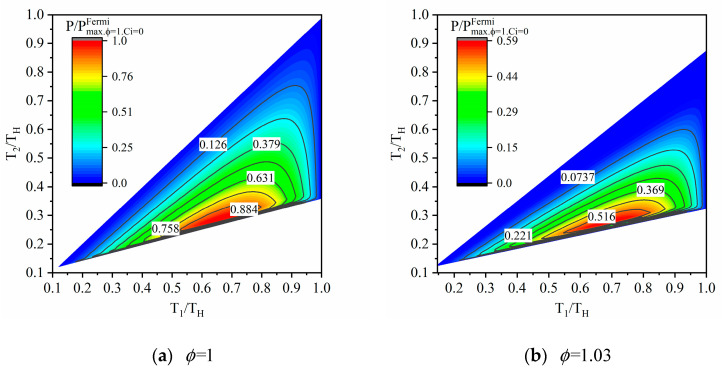
The output power of combined HE working with Fermi gas.

Figure 4 shows the relationship of dimensionless output power ($P/P_{\max}$) of combined HE working with Fermi gas versus dimensionless temperatures ($T_{1}/T_{H}$ and $T_{2}/T_{H}$) of WM, where $P_{\max.\phi =1.C_{i}=0}^{F\mathrm{ermi}}$ is the maximum output power of endoreversible combined Fermi HE (ϕ=1 and $C_{i}=0$). The horizontal coordinates and vertical coordinates are all dimensionless with the temperature of the hot reservoir ($T_{H}$). In this paper, the influence of the factor of entropy ratio (ϕ) on the output power is given instead of the heat leakage loss coefficient ($C_{i}$). The reason for this is that the heat leakage loss coefficient ($C_{i}$) does not affect the output power, according to Equation (32).

As can be seen from the temperature range of WM, the operating range of the combined heat engine is confined within a finite area. That is, when the high temperature of WM is fixed, the low temperature of WM is available in a small range that keeps the combined heat engine operates normally. In addition, there is a set of optimal temperatures of WM at which the combined heat engine output maximum power. According to Figure 4b, due to the effect of internal irreversibility, the operating temperature range of the combined cycle becomes smaller, and the dimensionless maximum output power decreases ($P/P_{\max.\phi =1.C_{i}=0}^{F\mathrm{ermi}}<1$).

**Figure 5:**
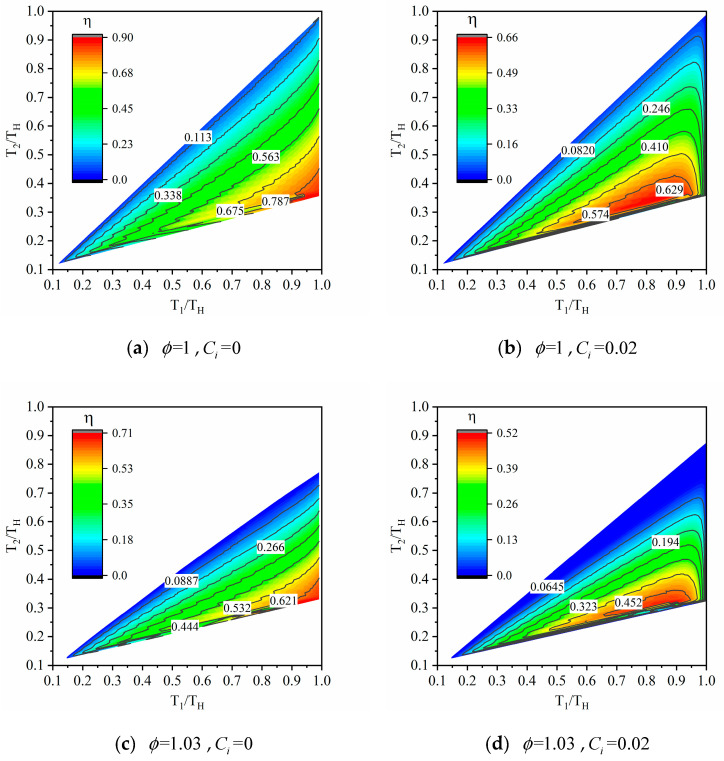
The thermal efficiency of the combined HE working with Fermi gas.

The relationship of thermal efficiency (η) of combined HE working with Fermi gas versus dimensionless temperatures ($T_{1}/T_{H}$ and $T_{2}/T_{H}$) of WM is depicted in Figure 5. The horizontal coordinates and vertical coordinates are all dimensionless with the temperature of the hot reservoir ($T_{H}$). It can be seen from the contour that there are two types of relationship between thermal efficiency and temperatures of WM. When there is no heat leakage loss ($C_{i}=0$), the thermal efficiency is directly proportional to the high temperature of WM and is inversely proportional to the low temperature of WM. When there is a heat leakage ($C_{i}=0.02$), there is a set of optimal temperatures ($T_{1}$,$T_{2}$) that makes thermal efficiency maximize. Both internal irreversibility and heat leakage loss weaken the thermal efficiency.

### 4.2. The Optimal Power Output and Thermal Efficiency

Equation (36) is adopted to solve the optimal performance of the combined HE with quantum gas. Since the Lagrangian function is very complex and nonlinear, the numerical method is utilized to solve in this paper.

Because of the shortcoming that the physical parameters of a single WM are fixed, different WMs are often used for improving performance in the combined HEs [40,41]. Since several sub-cycles are involved in the combined HE, it will contribute to improved performance of combined cycles if the suitable types of WMs are chosen to match the characteristics of cycles. Therefore, when calculating the optimal performance of the combined cycle, four types of WMs, including Fermi gas, Bose gas, Fermi–Bose gas (the WM of top sub-cycle is Fermi gas and the WM of bottom sub-cycle is Bose gas), and Bose–Fermi gas (the WM of top sub-cycle is Bose gas and the WM of bottom sub-cycle is Fermi gas), are compared in this paper.

**Figure 6:**
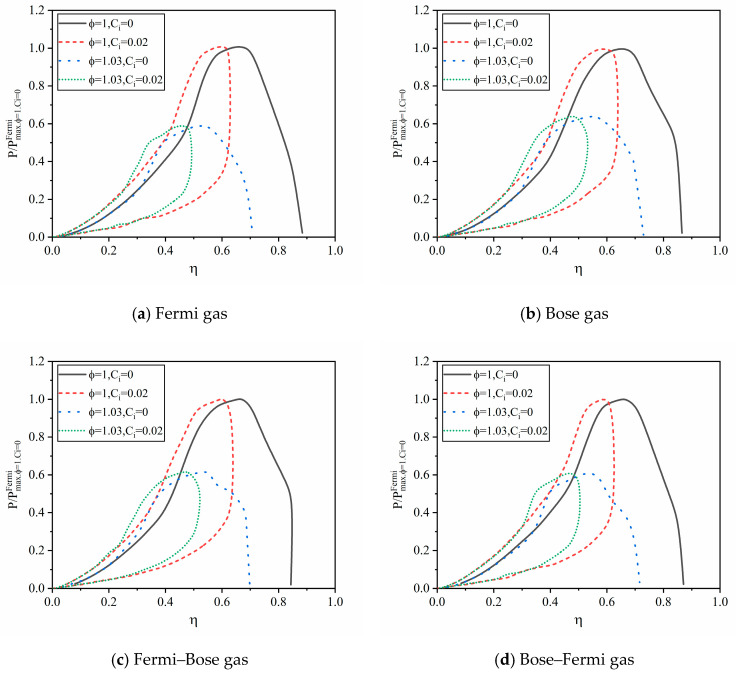
The optimal output power versus thermal efficiency of the combined HE with different quantum WM.

Figure 6 depicts the relationship of the optimal power versus thermal efficiency with four types of WMs. For four types of WMs, all the relationships of the optimal power and thermal efficiency are similar and appear two types of curves. When there is no heat leakage loss ($C_{i}=0$), the characteristic curve of power and thermal efficiency is a parabolic-like one. When there is internal irreversibility, i.e., ϕ>1, both power and thermal efficiency decrease. When there is heat leakage loss ($C_{i}>0$), the characteristic curve of power and thermal efficiency is a loop-shaped one. The heat leakage loss weakens the thermal efficiency of the combined HE, but does not affect the power, which can also be seen from Equations (32) and (33). In terms of the relationship between optimal power output and efficiency, the combined Carnot engine and the standard Carnot engine are the same. But for the combined HE working with quantum gas, the operating range of the bottom sub-cycle is constrained, and the operating conditions of the bottom sub-cycle are determined by the WM temperature and the thermal conductance distribution of the top sub-cycle. What’s more, the different types of quantum gases also affect power out and efficiency. The comparison of the two types of quantum gases will be given in the following.

Table 1 lists optimal output power and optimal thermal efficiency of the endoreversible combined HE with four types of WMs. Both maximum output power and thermal efficiency of the combined Fermi HE are superior to that of the combined Bose HE. In other words, higher power output can be achieved by using Fermi gas as WM in the high-temperature region, and higher power output can be achieved by using Bose gas as WM in the low-temperature region. When two types of quantum gases are selected for the WM, the combined HE working with Fermi–Bose gas obtains the highest output power. In fact, in order to simplify the calculation, this paper assumed that the entropy ratios of the four irreversible processes of the combined Carnot cycle were equal ($\phi_{1}=\phi_{2}=\phi_{3}=\phi_{4}=\phi$), so this result may be related to the entropy ratio of the irreversible process. In addition, the gas physical properties of the two kinds of quantum gases are different, which may also affect the power output. Therefore, more theoretical analysis and experiments are needed to verify the results of this paper.

It should be noted that the maximum efficiency of the combined HE is still equal to the Carnot efficiency. According to Equation (33), when the combined cycle is endoreversible (ϕ=1, $C_{i}=0$), the thermal efficiency reaches the highest and is given by

(38) $$\eta_{\max}=1-\frac{T_{2}}{T_{1}}\cdot \frac{T_{4}}{T_{3}}$$

In the limiting case, the temperatures of WM meet $T_{2}=T_{3}$, $T_{1}=T_{H}$, and $T_{4}=T_{L}$, Equation (38) is rewritten as

(39) $$\eta_{\max}=1-\frac{T_{L}}{T_{H}}=\eta_{C}$$

According to the calculating example in this paper, it can be seen from Table 1 that the maximum efficiency is in a range from 0.877 to 0.884 which is close to the Carnot efficiency ($\eta_{C}=1-10K/90K=0.89$). Considering the error caused by numerical calculation, it can be concluded that the maximum efficiency is equal to the Carnot efficiency.

## 5. Discussions

Under some special conditions, the expressions for power and thermal efficiency can be further simplified.

### 5.1. The Weak Degeneracy Condition

When the temperature of the WM is very low or the density of quantum gas is very low, it is a weak degeneracy condition. The corresponding Fermi integral and Bose integral can be expanded in the power of z. For the first-order approximation, the two correction factors of quantum gases are written as $F\left(T,v\right)=1\pm B/\left(T^{3/2}v\right)$, where the sign ‘±’ corresponds to ideal Fermi gas and Bose gas.

The natural logarithm of the corresponding fugacity is simplified as $\ln z=\ln\left[4\sqrt{2}B/\left(T^{3/2}v\right)\right]\pm 2B/\left(T^{3/2}v\right)$, then entropy can be denoted as

(40) $$S=\frac{5}{2}-\ln\frac{4\sqrt{2}B}{T^{3/2}v}\pm \frac{B}{2T^{3/2}v}$$

where $B=h^{3}/\left[16g{\left(mk\pi \right)}^{3/2}\right]$.

At this condition, Equations (19) and (20) are further simplified as

(41) $$D_{1}=\frac{\frac{5}{2}-\ln\frac{4\sqrt{2}B}{T_{2}{}^{3/2}v_{2}}\pm \frac{B}{2T_{2}{}^{3/2}v_{2}}-\left(\frac{5}{2}-\ln\frac{4\sqrt{2}B}{T_{1}{}^{3/2}v_{1}}\pm \frac{B}{2T_{1}{}^{3/2}v_{1}}\right)/\phi_{1}}{\left(\frac{5}{2}-\ln\frac{4\sqrt{2}B}{T_{2}{}^{3/2}v_{2}}\pm \frac{B}{2T_{2}{}^{3/2}v_{2}}\right)/\phi_{2}-\left(\frac{5}{2}-\ln\frac{4\sqrt{2}B}{T_{1}{}^{3/2}v_{1}}\pm \frac{B}{2T_{1}{}^{3/2}v_{1}}\right)}$$

(42) $$D_{2}=\frac{\frac{5}{2}-\ln\frac{4\sqrt{2}B}{T_{4}{}^{3/2}v_{4}}\pm \frac{B}{2T_{4}{}^{3/2}v_{4}}-\left(\frac{5}{2}-\ln\frac{4\sqrt{2}B}{T_{3}{}^{3/2}v_{3}}\pm \frac{B}{2T_{3}{}^{3/2}v_{3}}\right)/\phi_{3}}{\left(\frac{5}{2}-\ln\frac{4\sqrt{2}B}{T_{4}{}^{3/2}v_{4}}\pm \frac{B}{2T_{4}{}^{3/2}v_{4}}\right)/\phi_{4}-\left(\frac{5}{2}-\ln\frac{4\sqrt{2}B}{T_{3}{}^{3/2}v_{3}}\pm \frac{B}{2T_{3}{}^{3/2}v_{3}}\right)}$$

Substituting Equations (41) and (42) into Equations (32) and (33) can yield the simplified expressions of power and thermal efficiency.

### 5.2. The Strong Degeneracy Condition

When the temperature of the WM is very low and the density of quantum gas is very high, the Fermi integral can be described by an approximate expression. At this condition, the Fermi integral ($f_{l}\left(z\right)=\frac{1}{\Gamma \left(l\right)}\int_{0}^{\infty }\frac{x^{l-1}}{z^{-1}e^{x}+1}dx$) can be expanded in power of ${\left(\ln z\right)}^{-1}$. For the first-order approximation, the correction factor and natural logarithm of the corresponding fugacity are written as $F\left(T,v\right)=2T_{F}/5T+\pi^{2}T/6T_{F}$ and $\ln z=T_{F}/T-\pi^{2}T/12T_{F}$, respectively. So the entropy can be simplified as

(43) $$S=Nk\frac{\pi^{2}T}{2T_{F}}$$

where $T_{F}=A/v^{2/3}$ is Fermi temperature, $A={\left(3h^{3}\right)}^{2/3}/\left(8\pi^{2/3}km\right)$.

At this condition, Equations (19) and (20) are further simplified as

(44) $$D_{1}=\left(T_{2}v_{2}{}^{2/3}-T_{1}v_{1}{}^{2/3}/\phi_{1}\right)/\left(T_{2}v_{2}{}^{2/3}/\phi_{2}-T_{1}v_{1}{}^{2/3}\right)$$

(45) $$D_{2}=\left(T_{4}v_{4}{}^{2/3}-T_{3}v_{3}{}^{2/3}/\phi_{3}\right)/\left(T_{4}v_{4}{}^{2/3}/\phi_{4}-T_{3}v_{3}{}^{2/3}\right)$$

Substituting Equations (44) and (45) into Equations (32) and (33) can yield the simplified expressions of power and thermal efficiency.

## 6. Conclusions

An irreversible combined Carnot cycle model utilizing ideal quantum gas as WM is established in this paper. Under the irreversible conditions of thermal resistance, internal irreversibility and heat leakage loss, the output performance of the combined HE is analyzed and optimized. The main results are as follows:

(1) According to the exhausting heat of the top sub-cycle, the operating range of the bottom sub-cycle is constrained, and the operating conditions of the bottom sub-cycle can be determined by the WM temperature and the thermal conductivity distribution of the top sub-cycle.
(2) There is a set of optimal temperatures ($T_{1}$,$T_{2}$) that makes output power maximize. When there is heat leakage loss, there is also a set of optimal temperatures ($T_{1}$,$T_{2}$) that makes thermal efficiency maximize.
(3) When there is no heat leakage loss ($C_{i}=0$), the characteristic curve of power and thermal efficiency is a parabolic-like one. When there is heat leakage loss ($C_{i}>0$), the characteristic curve of power and thermal efficiency is a loop-shaped one. The internal irreversibility makes both power and thermal efficiency decrease. The heat leakage loss weakens the thermal efficiency of the combined HE, but does not affect the power.
(4) Under the assumption that the entropy ratios of the four irreversible processes of the combined Carnot cycle were equal, the optimal power and optimal efficiency of the combined Fermi HE is superior to that of the combined Bose HE. When two types of quantum gases are selected for the WM, the combined HE working with Fermi–Bose gas obtains the highest output power.

## Figures and Tables

**Table 1:** Optimal performance of the endoreversible combined HE working with four types of gases.

	Fermi Gas	Bose Gas	Fermi–Bose Gas	Bose–Fermi Gas
$P_{\max.\phi =1.C_{i}=0}/P_{\max.\phi =1.C_{i}=0}^{F\mathrm{ermi}}$	1	0.997	1.004	1.001
$\eta_{\max}$	0.884	0.877	0.878	0.883

## Data Availability

The data presented in this study are available on request from the corresponding author. The data are not publicly available due to privacy.

## Nomenclature

A	heat transfer area, $m^{2}$
C	heat leakage coefficient, W/K
CF	correction factor
D	internal irreversibility
g	number of possible spin orientations of a gas particle
h	Planck’s constant, J⋅s
k	Boltzmann’s constant, J/K
L	Lagrangian function
m	rest mass of a gas particle, kg
n	number density of gas particles, ${1/m}^{3}$
N	total number of particles
P	power output, W
p	gas pressure, Pa
Q	amount of heat exchange, J
$\dot{Q}$	rate of heat flow, W
S	entropy, J/K
t	heat transfer time, s
T	temperature, K
U	internal energy, J
V	volume of gas, $m^{3}$
v	average volume that an ideal quantum gas particle occupies, $m^{3}$
W	work, J
Ζ	simplified factor
z	fugacity of gas
**Greek Letters**	
α	heat transfer coefficient, $W/\left(m^{2}\cdot K\right)$
ϕ	entropy ratio
Γ	gamma function
η	efficiency
μ	chemical potential, J
τ	cycle period, s
**Subscripts**	
B	Bose gas
F	Fermi gas
H	hot side
i	heat leakage
L	cold side
q	ideal quantum gas
0	condensed state
1,2,3,4	cycle states
**Superscripts**	
′	The irreversible process
**Abbreviations**	
EMP	efficiency at the maximum power
HE	heat engine
MPO	maximum power output
QHE	quantum heat engine
WM	working medium
